# The Vicious Cycle of Renal Lipotoxicity and Mitochondrial Dysfunction

**DOI:** 10.3389/fphys.2020.00732

**Published:** 2020-07-07

**Authors:** Mengyuan Ge, Flavia Fontanesi, Sandra Merscher, Alessia Fornoni

**Affiliations:** ^1^Katz Family Division of Nephrology and Hypertension, Department of Medicine, University of Miami Miller School of Medicine, Miami, FL, United States; ^2^Peggy and Harold Katz Family Drug Discovery Center, University of Miami Miller School of Medicine, Miami, FL, United States; ^3^Department of Biochemistry and Molecular Biology, University of Miami Miller School of Medicine, Miami, FL, United States

**Keywords:** kidney, glomerular diseases, lipotoxicity, mitochondrial dysfunction, oxidative stress

## Abstract

The kidney is one of the most energy-demanding organs that require abundant and healthy mitochondria to maintain proper function. Increasing evidence suggests a strong association between mitochondrial dysfunction and chronic kidney diseases (CKDs). Lipids are not only important sources of energy but also essential components of mitochondrial membrane structures. Dysregulation of mitochondrial oxidative metabolism and increased reactive oxygen species (ROS) production lead to compromised mitochondrial lipid utilization, resulting in lipid accumulation and renal lipotoxicity. However, lipotoxicity can be either the cause or the consequence of mitochondrial dysfunction. Imbalanced lipid metabolism, in turn, can hamper mitochondrial dynamics, contributing to the alteration of mitochondrial lipids and reduction in mitochondrial function. In this review, we summarize the interplay between renal lipotoxicity and mitochondrial dysfunction, with a focus on glomerular diseases.

## Introduction

Measurement of resting metabolic rates shows that the kidney and heart have the highest resting energy expenditure among major organs and tissues in adults (Wang et al., 2010). Mitochondria provide energy to support kidney functions such as removing waste and balancing fluid. The energy currency ATP is mostly generated by oxidative phosphorylation (OXPHOS) coupled to electron transport through the mitochondrial respiratory chain (MRC). Increasing evidence indicates that mitochondrial damage and dysfunction are major contributors to the pathogenesis of chronic kidney disease (CKD) (Che et al., 2014). Lipids serve as a source and reservoir of energy supply. However, lipid surplus can hamper mitochondrial dynamics, contributing to increased reactive oxygen species (ROS) production and mitochondrial dysfunction. Conversely, impaired mitochondrial function can disturb the balance between lipid availability and lipid use, further increasing lipid accumulation (Schrauwen-Hinderling et al., 2016). Excess renal lipids have been reported in CKD of different origin, suggesting the existence of renal lipotoxicity (Gai et al., 2019). Although it has been suggested that both lipid dysmetabolism and mitochondrial dysfunction contribute to CKD, it remains to be established whether renal lipotoxicity is the cause or the consequence of mitochondrial dysfunction in the pathogenesis of CKD.

While tubular cells are mainly responsible for fluid and waste balance and account for most of the energy metabolism in the kidney, mitochondrial dysfunction and lipotoxicity were recently also described in diseases of glomerular origin. This is of particular importance as, different from tubular cells, key glomerular cells forming the kidney filtration barrier such as podocytes are terminally differentiated and, once injured, are unlikely to be replaced, thus causing proteinuria and disease progression (Kriz et al., 1998). Unlike tubular epithelial cells which preferentially use fatty acid (FA) β-oxidation as the main energy source (Kang et al., 2015), podocytes, endothelial cells, and mesangial cells in glomeruli highly rely on glucose as the substrate for energy production, while FA is used as an alternative substrate (Abe et al., 2010; Fink et al., 2012; Czajka and Malik, 2016). In the diabetic kidney, glucose oxidation in glomerular cells is disrupted and could account for the switch to FA oxidation (Forbes and Thorburn, 2018). In support, Imasawa et al. reported an oxidative shift during podocyte differentiation, where the expression of most glycolytic enzymes is decreased while the expression of proteins that are involved in FA β-oxidation is up-regulated (Imasawa et al., 2017). Peroxisome proliferator-activated receptor (PPAR)-γ coactivator (PGC)-1α expression can also induce a shift from their baseline glucose preference toward FA usage in podocytes (Li et al., 2017). Glomerular diseases, either primary or secondary to systemic diseases, represent an important cause of CKD (D’Agati et al., 2011). While we previously discussed how lipid accumulation contributes to mitochondrial dysfunction (Ducasa et al., 2019a), the current review focusses on and highlights the “two-way” interaction between lipids and mitochondria. More precisely, we summarize the findings observed in the setting of glomerular diseases with the goal to link lipotoxicity and mitochondrial dysfunction to irreversible podocyte injury and glomerular diseases.

## Mitochondrial Function and Biogenesis

Mitochondria fulfill several pivotal roles in cellular metabolism. In aerobic organisms, mitochondria are mostly responsible for the conversion of nutrient-derived energy in the form of ATP molecules through the mitochondrion-housed pathways of the Krebs cycle, FA β-oxidation, and OXPHOS (Lenaz and Genova, 2010). The complete oxidation of sugars and fats to carbon dioxide is achieved in the Krebs cycle and is paired with the concomitant conservation of free energy in the form of the reducing equivalents, nicotinamide-adenine dinucleotide (NADH), and flavin-adenine dinucleotide (FADH_2_). The metabolic intermediate oxidized in the Krebs cycle, acetyl-coenzyme A (acetyl-CoA), derives from pyruvate generated by glycolysis in the cytosol and from the breakdown of FA. FA β-oxidation occurs primarily in mitochondria through a series of four enzymatic reactions that constitute the predominant energy-producing pathway in the kidney (Bhargava and Schnellmann, 2017). In mitochondria, both the Krebs cycle and FA β-oxidation generate NADH and FADH_2_, which in turn transfer electrons to the MRC. Here, energy is further converted into an electrochemical gradient across the mitochondrial inner membrane. This conversion is achieved by the coupling of electron transfer through the MRC components to the final electron acceptor, molecular oxygen, with proton translocation from the mitochondrial matrix to the intermembrane space. Lastly, the protein gradient is used as the source of energy to drive ATP synthesis. The MRC is formed by four multimeric complexes (CI–CIV) embedded in the mitochondrial inner membrane and two mobile electron carriers, coenzyme Q (CoQ) and cytochrome *c*. Together, the MRC and ATP synthase (or CV) constitute the mitochondrial OXPHOS system (Lenaz and Genova, 2010).

ROS are byproducts of mitochondrial respiration, generated by electron “leakage” to oxygen, mainly at the level of CI and CIII of the MRC, to form superoxide anion (Stowe and Camara, 2009). *In vivo*, superoxide radicals have a short half-life and are rapidly converted into hydrogen peroxide by the action of superoxide dismutases, SOD1 and SOD2, located in the mitochondrial intermembrane space and matrix, respectively. While at low levels, ROS fulfill physiological functions as signaling molecules, mitochondrial dysfunction is commonly associated with a cellular energy deficit and increased ROS production, leading to the oxidative damage of cellular structures (Vafai and Mootha, 2012). Free FA, when in excess, can act as mitochondrial respiration uncouplers and MRC CI inhibitors and can also cause oxidative stress. To avoid lipotoxicity, FA levels in mitochondria are tightly controlled. FAs are stored in cytosolic lipid droplets (LDs), which exhibit close physical and metabolic interactions with mitochondria (Aon et al., 2014).

Furthermore, mitochondrial dysfunction is also frequently characterized by structural and morphological alterations. Mitochondria form a complex and highly dynamic network inside cells, which is continuously shaped by the processes of mitochondrial fusion and fission (Lackner, 2014). Mitochondrial hyperbranching, fragmentation, and loss of the mitochondrial inner membrane invaginations, known as cristae, have been observed in pathological conditions associated with mitochondrial respiratory defects and stress (Vincent et al., 2016). In addition to their role in cellular energy production, mitochondria are a hub for anabolic reactions, since the Krebs cycle provides intermediates for a wide range of biosynthetic pathways. These include precursors for amino acids, nucleobases, the antioxidant molecule glutathione, and metal prosthetic group biosynthesis. Notably, the Krebs cycle intermediate citrate is exported into the cytosol, where it is used as a substrate for FA and cholesterol synthesis (Owen et al., 2002). Among lipids, the synthesis of cardiolipin (CL) takes place in mitochondria. CL is the signature lipid of the mitochondrial inner membrane, which has an essential structural role in cristae formation and is required for optimal MRC assembly and function (Claypool and Koehler, 2012).

Mitochondria are organelles of endosymbiotic origin and still retain their genome, the mitochondrial DNA (mtDNA), and gene expression machinery, the mitochondrial ribosomes. In humans, the mtDNA encodes for a complete set of tRNAs, the ribosomal RNAs, and a handful of proteins, all catalytic subunits of the OXPHOS system. It has been estimated that the mitochondrial proteome could comprise about 1,500 proteins (Vafai and Mootha, 2012). With only 13 polypeptides encoded by the mtDNA, most of these proteins, including the remaining subunits of the OXPHOS system, are encoded by nuclear genes, synthesized in the cytosol, and imported into mitochondria. Mutations in both nuclear and mitochondrial genes are causes of major energetic deficits associated with severe hereditary diseases known as mitochondrial disorders (Turnbull and Rustin, 2016). While defects in nuclear genes follow Mendelian inheritance, mutations in the mtDNA are maternally transmitted. Since each cell contains hundreds to thousands of mtDNA molecules, wildtype and mutant genomes can co-exist, a condition known as heteroplasmy. Clinical manifestation appears only when the fraction of mutated molecules reaches a certain threshold, which can be tissue-specific (Turnbull and Rustin, 2016). Primary mitochondrial disorders are multisystemic diseases, although high energy demanding organs, such as heart and brain, are frequently the most affected. Kidneys also require large amounts of energy, mainly for tubular reabsorption (Hansell et al., 2013). In several instances, renal manifestations have been reported in patients affected by mitochondrial disorders (Finsterer and Scorza, 2017). In addition to primary mitochondrial diseases, mitochondrial dysfunction has been shown to be involved in the etiology and progression of numerous human pathologies, including kidney diseases and, in particular glomerular diseases, which are the focus of this review.

## Major Glomerular Diseases Due to Mitochondrial Cytopathies

Genetic defects in mtDNA or nuclear DNA (nDNA) that encode mitochondrial proteins can result in mitochondrial cytopathies. Though most mitochondrial disorders with a renal phenotype are characterized by tubular dysfunction, mtDNA 3243A > G mutation and CoQ10 biosynthesis defects (due to nDNA mutation) are associated with two mitochondrial cytopathies primarily causing glomerular diseases, most commonly focal segmental glomerulosclerosis (FSGS).

### Mitochondrial DNA Mutations Associated With Glomerular Diseases

Thirteen proteins are encoded by the mitochondrial genome, all of which are subunits of the MRC (Anderson et al., 1981; Di Donato, 2009). Mutations in mtDNA cause defects in the mitochondrial OXPHOS, resulting in energy deficiency and increased ROS. The most common mtDNA point mutation 3243A > G in the tRNA^LEU^ gene is associated with mitochondrial encephalomyopathy, lactic acidosis, and stroke-like episodes (MELAS) syndrome. Representing one of the most frequent mitochondrial disorders, MELAS syndrome could be considered as an underlying cause of primary FSGS, as several studies reported glomerular manifestation, often presents as FSGS (Kurogouchi et al., 1998; Cheong et al., 1999; Hotta et al., 2001; Gucer et al., 2005; Rudnicki et al., 2016; Narumi et al., 2018). In patients with MELAS syndrome and renal symptoms, the renal disease initially manifests with proteinuria and typical changes of the glomerular basement membrane (GBM) (Kurogouchi et al., 1998; Cheong et al., 1999; Narumi et al., 2018). Biopsy samples from these patients show abnormal mitochondria in podocytes, as well as foot process effacement (Gucer et al., 2005; Narumi et al., 2018). Patients with other point mutations (m.A4269G, m.A5728G, and m.A5843G) that affect mitochondrial tRNAs present with various severe clinical phenotypes, from fatal cardiomyopathy to multi-organ failure. These mutations are not associated with MELAS but were also reported to be associated with an FSGS phenotype (Taniike et al., 1992; Scaglia et al., 2003; Meulemans et al., 2006). Interestingly, deafness was also described in individuals with MELAS syndrome, which resembles the symptom of another rare genetic disease characterized by mutations of another important constituent of the GBM, i.e., type IV collagen (Alport syndrome). Besides, FSGS has been reported in a case of Kearns-Sayre syndrome due to mtDNA deletion (Becher et al., 1999). It is worth noting that mtDNA mutations associated with glomerular diseases affect overall mitochondrial gene expression and, in consequence, cause combined MRC enzymatic deficiencies, commonly associated with very severe outcomes.

### Mutations in nDNA That Encodes Mitochondrial Proteins Associated With Glomerular Diseases

Besides the 13 proteins encoded by the mitochondrial genome, there are over 1,158 other nuclear-encoded mitochondrial proteins (Calvo et al., 2016). The enzymes of the CoQ10 biosynthetic pathway are encoded by nuclear *CoQ* genes. Located mainly in the mitochondrial inner membrane, CoQ10 is a carrier that transfers electrons from complex I and II to complex III of the MRC (Ernster and Dallner, 1995). At least 15 genes are required in CoQ10 biosynthesis (Desbats et al., 2015). Mutations in these genes may cause CoQ10 deficiency, some of which were reported to be associated with glomerular involvement. In humans, prenyl (decaprenyl) diphosphate synthase subunit 1 (PDSS1) and PDSS2 are responsible for the synthesis of the prenyl side chain of CoQ10 (Saiki et al., 2005). Deficiency in CoQ10 content was reported in a patient carrying compound heterozygous mutations in PDSS2. The patient was diagnosed with Leigh’s syndrome with nephropathy (Lopez et al., 2006). Dietary supplementation with CoQ10 was shown to rescue proteinuria and interstitial nephritis in Pdss2kd/kd mice (Saiki et al., 2008) and diabetic nephropathy in db/db mice (Sourris et al., 2012). Gasser et al. analyzed patients with FSGS and collapsing glomerulopathy for PDSS2 polymorphisms. Surprisingly, they found a reduction of CoQ10 content, irrespectively of the PDSS2 haplotype (Gasser et al., 2013). Vasta et al. also reported that one patient carrying a PDSS1 mutation presented with nephrotic syndrome (Vasta et al., 2012). Pathogenic CoQ2 variants are another cause of primary CoQ10 deficiency. Patients with CoQ2 mutations have been reported to exhibit nephropathy that can be treated by CoQ10 supplementation (Starr et al., 2018). Moreover, loss-of-function mutations in CoQ6 have been described as a cause of glomerular damage, and patients manifested with FSGS in renal biopsy (Heeringa et al., 2011). It is important to note that genetic testing in patients with steroid-resistant nephrotic syndrome identified mutations in several genes involved in mitochondrial function and CoQ10 biosynthesis, including AarF domain-containing kinase 4 (ADCK4), CoQ6, CoQ2, and PDSS2 (Lovric et al., 2016). Patients who carry mutations in the ADCK4 gene may present with CoQ10 deficiency and mitochondrial nephropathy manifesting as FSGS (Ashraf et al., 2013; Yang et al., 2018; Widmeier et al., 2020). ADCK4 interacts with members of the CoQ10 biosynthesis pathway and is involved in CoQ biosynthesis, although its function remains to be characterized.

## Mitochondrial Dysfunction Results in Renal Lipotoxicity

Mitochondrial dysfunction can lead to lipid metabolism disorders in several cell types. Vankoningsloo et al. studied preadipocytes incubated with inhibitors of mitochondrial respiration (antimycin A) and found that mitochondrial dysfunction induces triglyceride accumulation. This process is mediated by reduced PPARγ activity leading to decreased expression of muscle isoform of carnitine palmitoyltransferase I (CPT1) and FA β-oxidation. Direct conversion of glucose into triglycerides and activation of carbohydrate-responsive element-binding protein (ChREBP), a lipogenic transcription factor, were also observed (Vankoningsloo et al., 2005). Boren and Brindle observed that the induction of apoptosis leads to mitochondrial ROS generation in murine lymphoma cells and mouse embryo fibroblasts, which inhibits mitochondrial FA β-oxidation but increases acyl-CoA synthetase activity for FA synthesis. Thereby, the switch from FA β-oxidation into lipid synthesis results in LD accumulation in the cytoplasm (Boren and Brindle, 2012).

### Dysregulated Mitochondrial Lipid Utilization in Kidney Diseases

**Figure 1:**
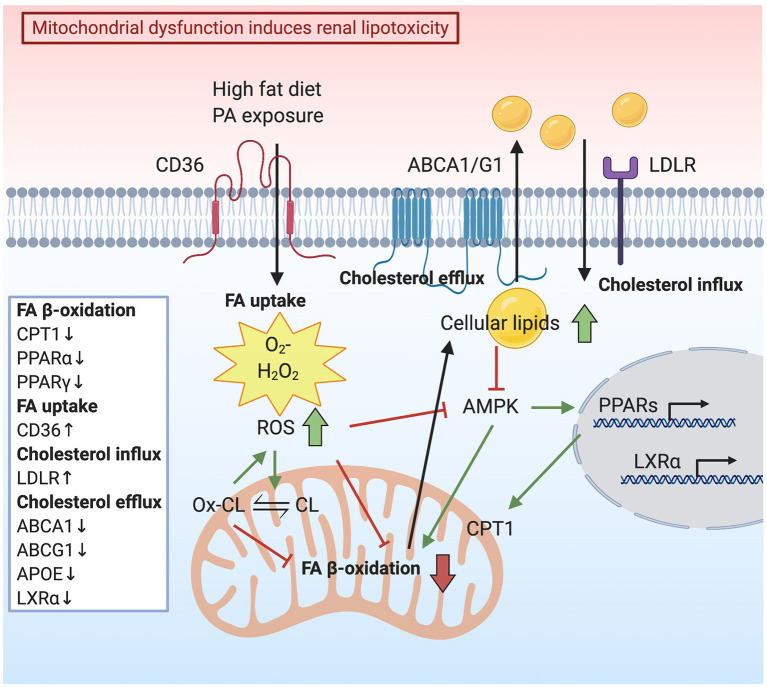
Schematic representation indicating how mitochondrial dysfunction induces renal lipotoxicity. High-fat diet or palmitic acid (PA) exposure increases mitochondrial reactive oxygen species (ROS) production. Consequently, elevated oxidative stress reduces mitochondrial fatty acid (FA) oxidation, resulting in lipid accumulation in the cytoplasm. Lipid accumulation is also caused by deceased cholesterol efflux mediated by ATP-binding cassette transporter A1 (ABCA1) and G1 (ABCG1) and elevated cholesterol influx *via* low-density lipoprotein receptor (LDLR). AMP-activated protein kinase (AMPK) suppression due to a lipid surplus inhibits mitochondrial FA oxidation and lipid utilization, accompanied by downregulation of transcription factors, liver X receptor (LXR) α, peroxisome proliferator-activated receptor (PPAR) α, and PPARγ. ROS generation contributes to cardiolipin (CL) peroxidation and loss of mitochondria cristae membranes, thus impairing the capacity for energy production.

Mitochondrial FA β-oxidation is the major energy pathway of the kidney. Mitochondrial dysfunction leads to compromised lipid utilization, eventually resulting in lipid accumulation, which is toxic to cells in the kidney. Herman-Edelstein et al. measured genes and enzymes involved in the FA β-oxidation pathway in kidneys of patients with diabetic nephropathy compared to healthy kidneys. The genetic analysis revealed the downregulation of some key transcriptional regulators of FA β-oxidation, such as PPARα, PPARγ, CPT1, and Aconitase 2 (ACO2) (Herman-Edelstein et al., 2014). Interestingly, upregulation of low-density lipoprotein receptor (LDLR) and cluster of differentiation 36 (CD36), downregulation of liver X receptor α (LXRα), ATP-binding cassette transporter A1 (ABCA1), ATP-binding cassette transporter G1 (ABCG1), and apolipoprotein E (APOE) were also detected, indicating alterations in cholesterol metabolism (Figure 1; Herman-Edelstein et al., 2014). In addition to diabetic nephropathy phenotypes including podocyte process effacement, thickening of the GBM, and mesangial expansion, electron microscopy analysis also showed extensive accumulation of LDs in podocytes, tubular epithelial cells, mesangial cells, and fenestrated endothelial cells (Herman-Edelstein et al., 2014). Consistently, CD36, PPARα, PPARγ, and LDLR were found to be genes with the highest connectivity to diabetic nephropathy progression in a study comparing human-mouse cross-species glomerular transcriptional networks (Hodgin et al., 2013).

A renal-protective effect of PPARs was also reported. The study by Calkin et al. suggests that the administration of PPARα, PPARγ, and PPARα/γ agonists in experimental mouse models of diabetes has renoprotective effects (Calkin et al., 2006). Doxorubicin is an antitumor drug that induces renal injury, leading to proteinuria. Doxorubicin-induced PPARα knockout mice showed more severe podocyte foot process effacement compared with Doxorubicin-induced wildtype mice but the treatment with PPARα agonist fenofibrate reduced proteinuria and ameliorated podocyte foot process effacement (Zhou et al., 2011). Fenofibrate has also been shown to prevent high-fat diet (HFD)-induced glomerular injury in an experimental model of diabetic nephropathy (Tanaka et al., 2011). Fenofibrate treatment reduces glomerular lipid (mainly triglycerides) accumulation, attenuates oxidative stress in kidneys of HFD-fed mice, and enhances the expression of lipolytic enzymes, including CPT1. Fenofibrate also attenuates tubulointerstitial injury by enhancing renal lipolysis (Tanaka et al., 2011). In the Fenofibrate Intervention and Event Lowering in Diabetes (FIELD) trial, a trend to renoprotection was observed in fenofibrate treated patients with diabetic nephropathy (Keech et al., 2005). PPARγ is also involved in renal lipid metabolism. In fact, PPARγ expression is reduced in glomeruli of diabetic mice, while activation of PPARγ inhibits the development of diabetic glomerular lesion (Zheng et al., 2002). Moreover, another study demonstrated that both PPARα and PPARγ agonists increase LXRα and ABCA1 gene expression and enhance apolipoprotein A1 (APOA1)-mediated cholesterol efflux from human mesangial cells (Ruan et al., 2003). However, PPARγ2 KO mice generated in an ob/ob background exhibit lipid accumulation at both the glomerular and tubular levels, accompanied by glomerular damage, in addition to the original metabolic syndrome phenotype (Martinez-Garcia et al., 2012). Located in the mitochondrial inner membrane, uncoupling proteins (UCPs) are mitochondrial transporters that control the level of coupled respiration (Rousset et al., 2004). Ke et al. reported that UCP2 expression is increased in human biopsy samples and mice kidney tissues with tubulointerstitial fibrosis but UCP2 deficient mice display reduced lipid accumulation and attenuated hypoxia in kidney tissue. Compared with wildtype mice, the expression of PPARα and CPT1α, which are the major regulators of lipid metabolism, is restored in UCP2 deficient mice (Ke et al., 2020). Both CPT1 and ACO2 are the rate-limiting enzymes of FA β-oxidation. Idrovo et al. demonstrated that stimulating CPT1 activity with a synthetic compound (C75) not only restores ATP generation but also improves renal function using a rat model of ischemia/reperfusion injury (Idrovo et al., 2012). In addition, mRNA levels of ACO1 and ACO2 are decreased in the tubulointerstitial and the glomerular compartment of non-diabetic CKD patients when compared to healthy controls (Hallan et al., 2017).

### Oxidative Stress Causes Renal Lipid Accumulation

Free FAs are sources of energy to synthesize ATP by mitochondrial FA β-oxidation. Palmitic acid (PA) is the predominant circulating saturated FA, which plays an important role in podocyte injury through lipotoxicity initiated by mitochondrial superoxide overproduction (Lee et al., 2017). Podocytes are vulnerable to PA-induced oxidative damage due to impaired peroxidase activity. Podocytes incubated with PA show increased FA uptake, mitochondrial superoxide, and hydrogen peroxide (H_2_O_2_) generation, as well as elevated AMP-activated protein kinase (AMPK) α phosphorylation (Figure 1; Lee et al., 2017). Furthermore, Liu et al. demonstrated that PA induces intracellular lipid accumulation and LD formation. It also induces podocyte apoptosis through activation of the mitochondrial pathway and triggers the release of cytochrome *c* from the mitochondria to the cytosol (Liu et al., 2018). In mouse podocytes, PA induces apoptosis by increasing cytosolic Ca^2+^ concentration, resulting from Ca^2+^ accumulation in the mitochondria *via* the mitochondrial Ca^2+^ uniporter (Yuan et al., 2017). The depletion of endoplasmic reticulum (ER) Ca^2+^ is also due to oxidative stress caused by PA (Xu et al., 2015). Under diabetic conditions, podocytes are susceptible to PA-induced oxidative damage due to an inadequate activity of peroxidase, the enzyme that catalyzes the conversion of the intracellular H_2_O_2_ to water in response to excess ROS generation (Lee and Lee, 2018). On the contrary, the nontoxic mono-unsaturated FA oleic acid (OA) inhibits the PA-induced ROS formation in podocytes (Lee et al., 2017; Lee and Lee, 2018). OA attenuates ROS generation and protects the mitochondria from PA-induced oxidative stress by the restoration of AMPK activity (Palomer et al., 2018).

Oxidative stress in the form of lipid peroxidation can be caused by the generation of lipid peroxyl radicals in podocytes. Excessive accumulation of lipid peroxyl radicals contributes to podocyte injury in CKD (Kruger et al., 2018). It was demonstrated that ROS generation contributes to glomerular and tubular injury through lipid peroxidation of cell and organelle membranes. ROS-induced lipid peroxidation disrupts the structural integrity of the lipid bilayer and impairs the capacity for energy production (Baud and Ardaillou, 1993). SS-31 is a tetrapeptide that protects mitochondria cristae structure and matrix density in all kidney cells. SS-31 targets CL, the phospholipid almost exclusively present in the inner mitochondrial membrane (Szeto and Birk, 2014). CL peroxidation alters mitochondrial cristae formation (Szeto, 2014), thus disrupting the functional and structural integrity of respiratory supercomplexes (Pfeiffer et al., 2003). Moreover, during apoptosis, CL binds to cytochrome *c* to form peroxidase complexes capable of catalyzing CL peroxidation and promoting cytochrome *c* release from mitochondria (Kagan et al., 2005). SS-31 selectively binds to CL, preventing CL from converting cytochrome *c* into a peroxidase while maintaining its function as an electron carrier (Szeto, 2014). The study of Szeto et al. described changes in mitochondrial structure in glomerular endothelial cells, podocytes, and proximal tubular epithelial cells after HFD induction. HFD increases mitochondrial ROS generation, causing CL peroxidation and loss of mitochondria cristae membranes. Increased ROS limits mitochondrial FA β-oxidation, which in turn causes cellular lipid accumulation and inhibits AMPK activity (Figure 1; Szeto et al., 2016). By preserving normal mitochondrial cristae structure, SS-31 treatment was able to restore AMPK activity and prevent intracellular lipid accumulation, ER stress, and glomerular inflammation (Szeto et al., 2016). AMPK is a cellular energy sensor that regulates glucose, lipid, and protein metabolism. During energy shortage, AMPK is activated and promotes ATP production (Hardie et al., 2012). On the contrary, AMPK is down regulated under conditions of energetic oversupply (Picard et al., 2012). Mice fed on a HFD show increased glomerular area and matrix accumulation. A significant rise of cholesteryl esters and phospholipids in the kidneys was also observed, in association with reduced AMPK activity. Reduced AMPK activity was also described in kidneys from both diabetic mice and in patients with diabetes (Dugan et al., 2013). Conversely, the activation of AMPK reverses the accumulation of cholesteryl esters and phospholipids in kidneys of mice fed a HFD (Decleves et al., 2011, 2014). Improving mitochondrial biogenesis and function by AMPK activation was found to be a therapeutic approach in a rat model of diabetic nephropathy (Zhou et al., 2019). The administration of AMPK activators increased the expression of PGC-1α, which induces a ROS scavenging mechanism, including the expression of SOD2 in the presence of ROS, protecting cells against ROS generation and damage (Spiegelman, 2007; Zhou et al., 2019). Similarly, AMPK activation in diabetic mice restores mitochondrial content and function while improving DKD progression (Figure 1; Dugan et al., 2013).

## Inherited Lipid Disorders are Associated with Renal Injury

Defects in genes relevant to lipid metabolism result in inherited diseases associated with renal lipotoxicity. Tangier disease is a rare disorder characterized by reduced levels of high-density lipoprotein (HDL) in the blood. Patients with Tangier disease have mutations in the ABCA1 gene and present with mild proteinuria and foamy podocytes in kidney biopsy (Ferrans and Fredrickson, 1975; Schaefer et al., 2010). Similarly, foamy podocytes, vacuolated tubular epithelial cells, and interstitial foam cells were described in a case report of a patient with Niemann-Pick disease, an inherited condition with an accumulation of sphingomyelin in various organs, including kidneys (Grafft et al., 2009). Caused by the accumulation of cholesterol in blood and tissue, familial lecithin-cholesterol acyltransferase (LCAT) deficiency is another genetic disorder, where some patients present with kidney injury. Renal biopsies of patients with LCAT deficiency identified LDs in mesangial and endothelial cells (Ossoli et al., 2015). Borysiewicz et al. described that patients with LCAT deficiency show disturbance of lipid metabolism and accumulation of lipids in the kidneys, eventually leading to end-stage kidney disease (Borysiewicz et al., 1982). In addition, transgenic mice with LCAT deficiency develop proteinuria and glomerulosclerosis characterized by the accumulation of free cholesterol and polar lipids in glomeruli (Lambert et al., 2001). Disturbed lipid metabolism was also reported in rare cases of lipoprotein glomerulopathy. Patients with an APOE gene mutation are characterized by proteinuria and hyperlipidemia. It has been suggested that the mutation of APOE increases the affinity of lipoproteins for glomerular capillary walls and promotes the formation of lipoprotein aggregates (Sam et al., 2006; Tsimihodimos and Elisaf, 2011). The evidence that genetic impairment of genes regulating cholesterol efflux leads to glomerular disease strongly suggests a causative link between altered renal lipid metabolism and kidney disease. This is very different from genetic disorders of hyperlipidemia, such as familial hypercholesterolemia (FH), where the high level of circulating lipids does not necessarily translate into an increased accumulation of renal lipids and does not always cause a renal phenotype. While this remains controversial, a recent association between FH and CKD was reported (Emanuelsson et al., 2018), and a patient with homozygous FH was found to develop FSGS (Elmaci et al., 2007). Similarly, the accumulation of lipids was found in the renal parenchyma of patients affected by FH (Buja et al., 1979). Although some of these studies suggest a link between systemic hyperlipidemia and CKD, most of the studies have demonstrated that statins may partially reduce proteinuria but this does not result in the prevention of CKD progression (Agarwal, 2006).

## Glomerular Lipid Accumulation Results in Mitochondrial Dysfunction

Podocytes rely on a constant supply of lipids and proteins to form foot processes (Simons et al., 1999; Fornoni et al., 2014). However, lipid overload causes lipotoxicity. Lipid accumulation and increased inflammation accelerate lipotoxicity induced glomerular disease (Martinez-Garcia et al., 2015). Among glomerular cells, podocytes are terminally differentiated cells that are most susceptible to damage caused by lipid overload. The podocyte slit diaphragm is assembled as a lipid-raft like structure, enriched with lipids including sphingolipids and cholesterol. Plasma membrane and intracellular lipids can affect podocyte function (Fornoni et al., 2014). Localized to lipid raft domain, sphingomyelinase-like phosphodiesterase 3b (SMPDL3b) is a sphingolipid-related enzyme that modifies the plasma lipid composition and modulates intracellular inflammatory pathways in podocytes (Heinz et al., 2015; Yoo et al., 2015). Moreover, cholesterol is required for the proper function of the slit diaphragm (Simons et al., 2001), while excessive accumulation of cholesterol causes podocyte injury and proteinuria (Merscher-Gomez et al., 2013; Pedigo et al., 2016).

### Hyperlipidemia and Hyperlipidemia-Induced Lipotoxicity

**Figure 2:**
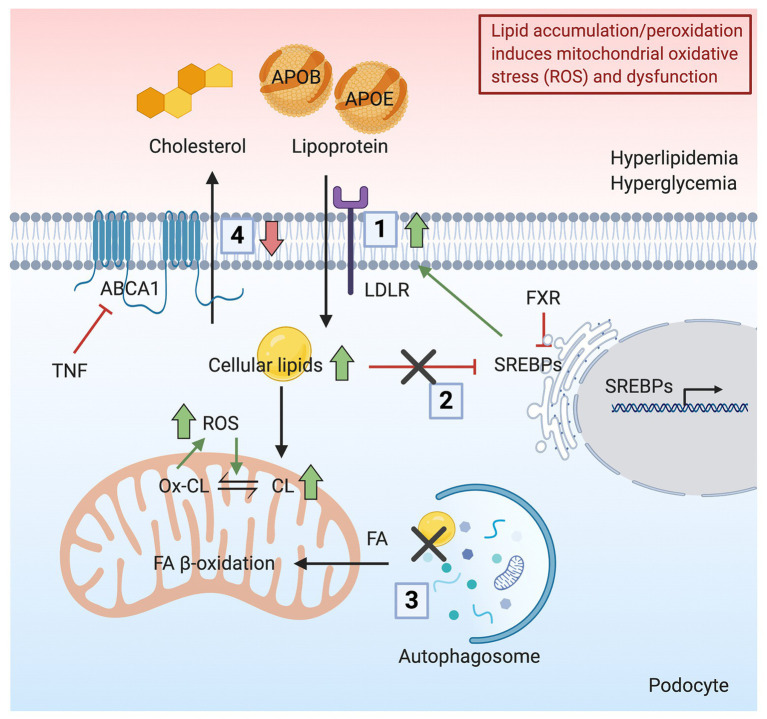
Schematic representation indicating how lipid accumulation/peroxidation induces mitochondrial oxidative stress and dysfunction. Podocyte lipid accumulation and increased peroxidation induces ROS production and mitochondrial dysfunction. (1) In the presence of hyperlipidemia, increased uptake of apolipoprotein B (APOB)‐ and apolipoprotein E (APOE)-rich lipoproteins occurs in podocytes through the LDLR, resulting in a lipid-rich environment. (2) Hyperglycemia disrupts the feedback regulation of sterol regulatory element-binding proteins (SREBPs), causing further lipid accumulation. (3) Cellular lipids, mainly in the form of triglycerides, stored in lipid droplets (LDs) are hydrolyzed and delivered as FAs to mitochondria by autophagic clearance. The inhibition of autophagic clearance of lipids is associated with increased lipid endocytosis. (4) Downregulation of ABCA1 impairs reverse cholesterol transport, resulting in the accumulation of cholesterol and CL and peroxidation of CL. Together, the accumulation of lipids in podocytes induces ROS production and mitochondrial dysfunction.

Foam cells are macrophages that ingest low-density lipoprotein (LDL) during hyperlipidemia. Their formation represents the hallmark of atherosclerosis (Yu et al., 2013). Interestingly, foam cells also appear in various glomerular diseases, including diabetic nephropathy, FSGS, nephrotic syndrome, and Alport syndrome (Nolasco et al., 1985; Stokes et al., 2006; Wu et al., 2009; Eom et al., 2015), indicating lipid-mediated toxicity. However, it is not established whether foam cells generated during the pathogenesis of kidney diseases are renal resident cells or infiltrating macrophages from the circulation (Eom et al., 2015). It is certain that glomerular cells can uptake LDL. In fact, the activity of lipoprotein receptors in cultured epithelial cells of the human glomerulus was characterized in the early 1990s. Grone et al. demonstrated that cultured glomerular epithelial cells show lipoprotein receptor activity, which mediates the uptake of apolipoprotein B (APOB)‐ and APOE-rich lipoproteins, contributing to lipid accumulation and oxidative stress (Grone et al., 1990). In addition, the accumulation of APOB and APOE in immune deposits could lead to GBM damage (Figure 2). Heymann nephritis is a rat model of membranous nephropathy characterized by the formation of subepithelial immune deposits in the GBM (Heymann et al., 1959). In Heymann nephritis, Kerjaschki et al. demonstrated that antibodies targeting megalin (an LDL-related protein) inhibit megalin-mediated binding and clearance of APOE and APOB, leading to the accumulation of apolipoproteins in immune deposits. The lipid environment is associated with lipid peroxidation, injury of the GBM, and proteinuria (Kerjaschki et al., 1997). Oxidized LDL has also been found to directly damage podocytes *via* the chemokine ligand 16 (CXCL16), as well as induce ROS production in podocytes (Bussolati et al., 2005; Gutwein et al., 2009a). CXCL16 is a major scavenger receptor for oxidized LDL in human podocytes. Both glomerular levels of CXCL16 and oxidized LDL are increased in patients with diabetic nephropathy and membranous nephropathy (Gutwein et al., 2009a,b).

Experimental evidence also supports a role of lipid-lowering agents in the protection of kidney diseases. For example, treatment of patients with lipid-lowering agents such as the hydroxymethylglutaryl coenzyme A (HMG-CoA) reductase inhibitor atorvastatin was able to lower serum lipids and glomerular lesions but to a lesser extent than the blockage of the renal angiotensin system with quinapril. In fact, the renal protective effect of atorvastatin is associated with lipid-related and non-lipid-related effects (Blanco et al., 2005). Once more, however, the renal protective effects of statin treatment remain to be confirmed in patients affected by CKD. Interestingly, hypercholesterolemia associated with nephrotic syndrome can be ameliorated by inhibiting proprotein convertase subtilisin/kexin type 9 (PCSK9), a posttranscriptional regulator of the LDL receptor. PCSK9 targets LDLR for degradation and thereby reduces the clearance of LDL from the circulation (Shrestha et al., 2019). Inhibition of PCSK9 decreases the proportion of APOB-associated cholesterol (VLDL and LDL) and increases the proportion of HDL-associated cholesterol, producing a more protective lipoprotein profile (Haas et al., 2016). Currently, the monoclonal antibodies evolocumab and alirocumab are approved PCSK9 inhibitors for therapeutic use and are effective and safe in patients with mild to moderate CKD (Schmit et al., 2019). However, it would be important to study if PCSK9 inhibitors can also alleviate proteinuria and CKD progression in patients with advanced CKD, such as nephrotic syndrome. Studies in cultured vascular smooth muscle and endothelial cells suggest that inhibition of PCSK9 attenuates ROS production, while PCSK9 overexpression increases ROS generation in a concentration-dependent manner (Ding et al., 2015). The crosstalk between PCSK9 and mtDNA damage in vascular smooth muscle cells is thought to be partially mediated by mitochondria-derived ROS (Ding et al., 2016). Lastly, more studies are needed to assess the contribution of LDL apheresis to the treatment of proteinuric kidney disease, in order to gain a better understanding of whether the antiproteinuric effects are linked to LDL apheresis or related proteins.

### Intrarenal Lipid Accumulation in Glomerular Diseases

Lipid distribution among plasma, tissues, and cellular organelles can be altered by inflammatory stress (Ruan et al., 2009). In addition to the uptake of plasma lipids into cells *via* lipoprotein receptors, other factors that mediate lipid accumulation in glomerular cells, thus contributing to disease development and progression, have been described in several kidney diseases. Among them, we have demonstrated that glomerular TNF is a major driver of lipid dysmetabolism in FSGS (Pedigo et al., 2016). Our group reported the accumulation of cholesterol in kidney cortices in mouse models of diabetes (Ducasa et al., 2019b), Alport syndrome, and FSGS (Pedigo et al., 2016; Mitrofanova et al., 2018). Impaired ABCA1 mediated reverse cholesterol transport in a mouse model of FSGS was rescued by genetic ABCA1 overexpression. Similarly, treatment with hydroxypropyl-β-cyclodextrin (HPβCD) was found to reduce cholesterol accumulation in kidney cortices and was found to protect from renal failure in mouse models of FSGS, Alport syndrome, and DKD (Pedigo et al., 2016; Mitrofanova et al., 2018). Additionally, lipid accumulation is associated with the downregulation of ABCA1 in podocytes treated with sera from patients diagnosed with type 1 diabetes or type 2 diabetes (Pedigo et al., 2016; Ducasa et al., 2019b). Moreover, podocyte-specific deletion of ABCA1 renders mice susceptible to DKD progression, while the induction of ABCA1 ameliorates podocyte injury (Ducasa et al., 2019b).

Apart from failing to remove lipids from cells, lipid oversupply is another major cause of renal dysfunction (Figure 2). Sterol regulatory element-binding proteins (SREBPs) activate genes involved in the synthesis and uptake of cholesterol, FAs, triglycerides and phospholipids, endocytosis of LDL, and glucose metabolism (Edwards et al., 2000; Horton et al., 2002). In DKD, increased expression of SREBPs is associated with the accumulation of triglycerides and cholesterol in the kidney, resulting in glomerulosclerosis and proteinuria (Sun et al., 2002). Similarly, C57BL/6J mice fed on a HFD also show renal accumulation of triglycerides and cholesterol, accompanied by glomerulosclerosis due to SREBPs pathway activation (Jiang et al., 2005). Moreover, LDLR plays a role in the feedback system that modulates plasma and intracellular cholesterol homeostasis. The expression of LDLR is tightly regulated by SREBPs and SREBP cleavage-activating protein (SCAP). In the presence of high glucose or inflammatory stress, the LDLR feedback regulation is disrupted, causing intracellular cholesterol accumulation and podocyte injury (Zhang et al., 2015a,b). Interestingly, the up-regulation of hepatic PCSK9 was found to be modulated by SREBP-2 and SREBP-1c, which could furthermore dysregulate LDL cholesterol (Jeong et al., 2008; Rong et al., 2017).

### Lipid Burden and Mitochondrial Dysfunction

The turnover of lipids in podocytes regulates podocyte health. Serezani reported that the expression of phosphatase and tensin homolog (PTEN) inhibits phagocytosis, cell growth, and cytoskeletal remodeling (Serezani et al., 2012). The downregulation of PTEN is associated with increased lipid endocytosis in podocytes from patients with obesity-related glomerulopathy, in cultured mouse podocytes with PTEN knock-down and in mice with podocyte-specific knockout of PTEN, thus enhancing the uptake of lipids and causing podocyte injury and proteinuria through oxidative stress response (Shi et al., 2019). On the contrary, elevated autophagic sequestration of LDs was reported as an adaption to alcoholic liver disease (Eid et al., 2013). In the study of Sato et al., podocytes with a higher level of autophagy are more competent to remove proteins and lipids from cells, suggesting a tendency to better prognosis (Sato et al., 2006). Autophagy related 5 (ATG5) is a critical autophagy gene during nephrogenesis. Mutation of ATG5 in the kidney epithelium leads to FSGS in mice, accompanied by enhanced ROS generation, ER stress, and mitochondrial dysfunction (Kawakami et al., 2015). Interestingly, autophagy regulates lipid metabolism by autophagic clearance of cellular lipids stored as triglycerides in LDs, which are then hydrolyzed and delivered as FAs for energy production in mitochondria (Figure 2; Singh et al., 2009).

Lipid surplus induces a vicious cycle where the accumulation of lipids induces ROS production and mitochondrial dysfunction. Conversely, dysfunctional mitochondria may decrease the lipid oxidative capacity, which additionally increases the lipid surplus (Schrauwen-Hinderling et al., 2016). In a rat model of hypertension and proteinuria, increased metabolic stress was indicated by increased oxidized lipids derived from lipid breakdown in glomeruli at the early stage, causing decreased ATP and NADH levels. The change of metabolic aspects in glomeruli from these mice was associated with the activation of phosphoprotein-dependent signaling such as mTOR and AMPK (Rinschen et al., 2019). Steinberg et al. demonstrated that AMPK activity is suppressed by TNFα signaling, resulting in decreased FA β-oxidation and excess lipids in skeletal muscle (Steinberg et al., 2006). Notably, we previously showed that serum TNFR1 and TNFR2 levels are increased in patients with DKD and FSGS, whereas TNF levels are only increased in patients with DKD. More importantly, we demonstrated that glomerular TNF expression rather than systemic TNF causes free cholesterol accumulation and podocyte injury (Pedigo et al., 2016). High lipid availability induced ROS production can furthermore cause lipid oxidation (Schrauwen-Hinderling et al., 2016). Fibroblasts from patients with Tangier disease, who have an inherited ABCA1 mutation, show an increased content of the mitochondrial-specific phospholipid CL, while patients have reduced HDL levels in the blood (Fobker et al., 2001). Our group demonstrated that ABCA1 deficiency in human podocytes leads to CL accumulation and mitochondrial dysfunction. SS-31 has been used to scavenge ROS and stabilize CL (Zhao et al., 2004). The administration of an Abca1 inducer or elamipretide (SS-31) reduces CL peroxidation and ameliorates podocyte injury, indicating that ABCA1 deficiency causes CL accumulation and peroxidation, eventually leading to mitochondrial dysfunction (Figure 2; Ducasa et al., 2019b).

Reduction of renal parenchymal lipids ameliorates mitochondrial dysfunction, thus mitigating kidney injury. FSGS with podocyte injury and depletion can be mediated by mitochondrial oxidative damage in adjacent endothelial cells *via* endothelin-1 (EDN1) signaling. Podocyte-derived EDN1 can act on endothelial cells, which mediates oxidative stress and endothelial dysfunction *via* EDN1 receptor type A (EDNRA) activation, thus promoting podocyte apoptosis. In support of this observation, mitochondrial-targeted ROS scavengers and endothelin antagonists inhibit the release of EDN1, thus reducing mitochondrial oxidative stress and endothelial cell dysfunction, thereby preventing glomerulosclerosis (Daehn et al., 2014). In DKD, glomerular endothelial mitochondrial dysfunction is also associated with increased glomerular EDNRA expression and increased circulating EDN1. EDNRA antagonist treatment prevents mitochondrial oxidative damage in endothelial cells and ameliorates diabetes-induced glomerular injury, suggesting a crosstalk between podocytes and glomerular endothelial cells (Qi et al., 2017). Interestingly, EDN1 promotes lipid accumulation during the transformation of macrophage foam cells through the downregulation of ABCG1 and impaired HDL-mediated cholesterol efflux (Lin et al., 2011). It is possible that the inhibition of EDN1 signaling could promote reduced renal lipotoxicity in addition to suppressing oxidative stress. The farnesoid X receptor (FXR) is a bile acid-activated nuclear receptor that regulates lipid metabolism by modulating renal SREBP-1 activity. Since the SREBPs play a critical role in renal lipid accumulation, the administration of FXR agonists ameliorates renal triglyceride accumulation and regulates renal lipid metabolism in HFD-fed mice by modulating FA synthesis and oxidation, improving proteinuria and kidney injury (Wang et al., 2009). In addition, administration of the dual agonist of FXR and G protein-coupled membrane receptor Takeda G protein-coupled receptor 5 (TGR5) to mice decreases proteinuria and prevents mitochondrial impairments. This dual agonist modulates renal lipid metabolism and prevents renal lipid accumulation in mouse models of nephropathy associated with diabetes and obesity. Dual agonist treatment prevents inflammation, oxidative stress, and ER stress, as well as induces mitochondrial biogenesis and metabolism pathways (Wang et al., 2017, 2018). Similarly, Gai et al. also reported that FXR activation reduces glomerulosclerosis and tubulointerstitial injury in mice fed on a HFD. FXR agonist treated mice showed improved renal function in association with reduced lipid accumulation and reduced ROS. Additionally, FXR activation attenuated the decrease in autophagosomes in obese mice, maintaining effective energy production (Gai et al., 2016).

## Conclusion

Lipids are essential sources for mitochondrial energy production. However, abnormal lipid metabolism in many tissues or cell types has proven to be detrimental and to contribute to the pathogenesis and progression of several disorders. We first summarized glomerular diseases caused by inherited mitochondrial disparities, as well as by inherited lipid disorders, thus providing the genetic evidence in humans that both altered mitochondrial function and lipid metabolism may contribute to the pathogenesis of kidney diseases. We then elaborated on the independent impacts of lipotoxicity and mitochondrial dysfunction on kidney diseases, especially diseases characterized by glomerular injury. Finally, we reviewed the evidence that lipotoxicity can be the cause or the consequence of mitochondrial dysfunction. Lipids form the bilayer membrane structure of mitochondria. However, the accumulation of lipids induces excess ROS generation and increased mitochondrial oxidative stress. Meanwhile, accumulation and peroxidation of CL interrupt the normal structure of mitochondria and impair mitochondrial respiration and energy production (Figure 1). On the other hand, mitochondria utilize lipids as a substrate for FA β-oxidation in order to meet high energy demand. Increased ROS production, secondary to stress conditions, inhibits mitochondrial FA β-oxidation and lipid utilization, which in turn generates more ROS (Figure 2). It is clear that ROS play a critical role in the interplay of mitochondrial dysfunction and glomerular lipotoxicity. Interweaved by ROS production, a vicious cycle is formed by down-regulated FA β-oxidation, accumulation of intracellular lipids and peroxidation of lipids (especially CL). Therefore, identifying therapeutic targets that break this vicious cycle may offer novel promising therapeutic interventions for the cure of glomerular diseases.

## Author Contributions

MG and FF conceived and wrote the manuscript. SM and AF critically reviewed and improved the entire manuscript. All authors contributed to the article and approved the submitted version.

## Conflict of Interest

AF and SM are inventors on pending or issued patents (PCT/US2019/041730 and PCT/US2019/032215) aimed at diagnosing or treating proteinuric kidney diseases. They stand to gain royalties from the future commercialization of these patents. AF is vice-president of L&F Health LLC and is a consultant for ZyVersa Therapeutics, Inc. ZyVersa Therapeutics, Inc. has licensed worldwide rights to develop and commercialize hydroxypropyl-beta-cyclodextrin from L&F Research for the treatment of kidney disease. AF is founder of LipoNexT LLC. SM is a consultant for Kintai Therapeutics, Inc. and holds equity interest in L&F Research. AF and SM are supported by Hoffman-La Roche and by Boehringer Ingelheim.

The remaining authors declare that the research was conducted in the absence of any commercial or financial relationships that could be construed as a potential conflict of interest.
